# Effects of thermal stress on calf welfare*

**DOI:** 10.3168/jdsc.2023-0443

**Published:** 2023-11-17

**Authors:** Jennifer Van Os, Kimberly Reuscher, Bethany Dado-Senn, Jimena Laporta

**Affiliations:** Department of Animal and Dairy Sciences, University of Wisconsin–Madison, Madison, WI 53706

## Abstract

Cold and heat stress present welfare challenges for dairy calves. The consequences of thermal stress on biological functioning have been well documented, and many housing and management strategies have been evaluated to mitigate those detrimental impacts. In cold weather, mitigation strategies have largely focused on nutritional interventions or limiting heat loss with resources such as bedding or jackets. In hot weather, heat abatement strategies such as supplemental shade, increased environmental air exchange through passive ventilation, and forced air movement through mechanical ventilation have been evaluated. Recently in Wisconsin's continental climate, our group evaluated how 2 aspects of calf welfare—the needs for thermal comfort and social contact (i.e., pair or group housing vs. individual housing)—may align or conflict in winter and summer, respectively. In both seasons, calves pair-housed in outdoor hutches preferred social proximity. When 2 calves shared a hutch, the heat load was greater than for a single calf, which may be beneficial for thermal comfort in winter. In summer, the potential detriments from the additional heat load of 2 calves was mitigated with passive hutch ventilation, which calves preferred. Nonetheless, knowledge gaps remain regarding the impacts of thermal stress on calves' affective states, and much remains unknown about their preferences and motivations for specific thermal stress mitigation resources. Future research to address these gaps could improve our understanding of calf welfare and inform best practices for calf management.

## Graphical Abstract

**Figure:**
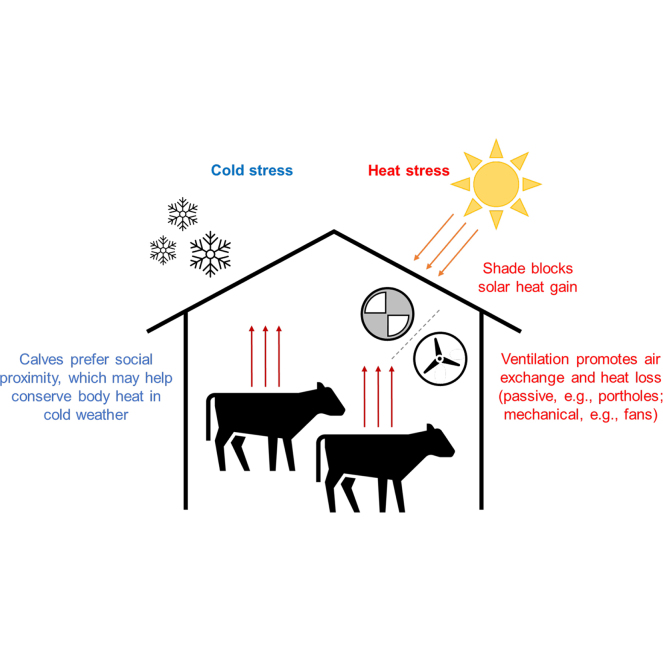

A common framework for studying animal welfare (Fraser et al., 1997) includes 3 overlapping categories of ethical values regarding an animal's biological functioning, internal affective (i.e., emotional) states, and ability to live a reasonably natural life (i.e., perform motivated behaviors). Decades of research have provided substantial knowledge about the biological functioning of dairy cattle, including measures of health, growth, production, and reproduction. Within applied ethology, techniques such as preference and motivation testing (Fraser and Nichol, 2011) are used to evaluate the value of certain resources or behaviors from the animal's perspective. The resulting inferences create opportunities to mimic the most important parts of natural living in confinement. In recent years, interest has grown in evaluating animals' subjective internal experiences to understand how to minimize negative affective states (e.g., pain, fear) and provide opportunities to promote positive ones (Paul and Mendl, 2018).

Studies of thermal stress in calves have primarily focused on biological functioning. Although some experiments have included behavioral responses, research is limited on calves' preferences in relation to thermoregulation, and no studies have evaluated affective states. Furthermore, few studies have considered thermal stress in the context of other important contributors to calf welfare, such as social contact. A growing body of literature (reviewed by Costa et al., 2016) has demonstrated positive impacts of social housing on calf welfare. From a natural living perspective, cattle are a social species; although preweaning calves are predominantly housed individually in the United States, they are motivated by (Ede et al., 2022) and prefer (Færevik et al., 2006) social contact and proximity with conspecifics. Social housing can promote positive affective states; calves raised in pairs, compared with those housed individually, showed more optimistic responses to ambiguous stimuli in judgment bias tests (Bučková et al., 2019). Numerous studies have shown that socially reared calves outperform individually housed counterparts in one or more outcomes relating to biological functioning, including solid feed intake, BW at weaning, or ADG (reviewed by Costa et al., 2016 and replicated in more recent studies, e.g., Knauer et al., 2021). Early-life social contact also facilitates nonphysical development, including cognitive flexibility (Meagher et al., 2015), reduced feed neophobia (Costa et al., 2014), and resilience to weaning stress (De Paula Vieira et al., 2010).

We posit that calves are motivated to seek both social contact and thermal comfort. Furthermore, we hypothesize these motivations could be aligned in cold weather because huddling provides social contact and promotes heat retention, and thus thermal comfort. In contrast, these motivations may conflict in warm weather, when social proximity may reduce heat dissipation, reducing thermal comfort. About 24% of US preweaning dairy calves are housed individually in outdoor hutches (USDA, 2021), where calves can be exposed to environmental extremes. Calves exchange heat with their surrounding environment, and when they are inside a hutch, some dissipated body heat may affect the hutch microclimate. In our recent work in Wisconsin's continental climate, we predicted that when 2 calves share the space, this would contribute to greater warming of the microclimate in both winter and summer. We also predicted that modifying the hutch to promote air exchange via natural ventilation could mitigate this effect in summer.

Cold stress presents a well-recognized challenge for calves, particularly neonatal ones. The lower critical temperature, the ambient temperature threshold below which they must expend energy to maintain homeothermy, is 10°C for dairy calves ≤8 wk of age (Webster et al., 1978). In much of North America, winter temperatures fall below this threshold, subjecting calves to seasonal cold stress, especially when housed outdoors and exposed to the additional effects of wind and precipitation. Calves are more susceptible to cold stress relative to older cattle because they have a larger surface area to mass ratio, resulting in greater heat loss, and a still-developing rumen that produces little heat associated with ruminal fermentation (Collier et al., 1982). In the last decades, several studies have documented the consequences of cold stress on biological functioning: maintenance requirements (Scibilia et al., 1987) and feed intake (Roland et al., 2016) increase, whereas growth performance decreases (Scibilia et al., 1987), immune function is suppressed (Olson et al., 1980b), and morbidity (Olson et al., 1980a) and mortality rates increase (Godden et al., 2005).

Strategies to mitigate the effects of cold stress in calves include nutritional interventions to meet the greater maintenance requirements (e.g., greater milk allowances, Anderson and Bates, 1984; fat supplementation in the liquid diet, Nonnecke et al., 2009) or methods to limit heat loss, such as by providing deep bedding (Nordlund, 2008) or calf jackets (Scoley et al., 2019). In a barn, 3-d-old calves have been shown to spend most of their time near heat lamps (Borderas et al., 2009). Calves raised in group pens in barns huddle together for warmth (Hänninen et al., 2003), with the magnitude of this behavior inversely proportional to ambient temperatures (B⊘e and Havrevoll, 1993). In a calorimetry study (Webster et al., 1978), the researchers noted heat loss was not significantly reduced when calves had the opportunity to huddle together; however, the effects of individual versus pair housing were not evaluated systematically, with calves kept in the calorimetry chamber “usually singly but occasionally in pairs.”

Our group recently evaluated the potential for social housing to counter the negative effects of cold stress in outdoor hutches in a continental winter (24-h mean air temperature: −2.1 ± 5.3°C; Reuscher et al., 2024a). Calves were housed individually or in pairs, with the latter hutches connected via the outdoor run (Figure 1A). We collected measures of not only biological functioning, but also calves' behavior and preferences; we found limited evidence that pair housing mitigates cold stress. All calves spent most of their time inside a hutch, but pair-housed ones spent more time outside than individually housed counterparts, perhaps reflecting their ability to recover heat more quickly once inside. In addition, calves within each pair preferred to spend 80% to 90% of their time together when in a hutch (Figure 2). When calves were enclosed inside a hutch for 1 h with a wire mesh panel, the internal hutch temperature increased further above baseline for pair- versus individually housed calves, although calf rectal temperature did not differ. After weaning, pair-housed calves tended to consume more starter concentrate than individually housed ones, but the treatments did not differ in ADG. Our study was the first evaluation of the potential for pair housing to enhance thermoregulation in calves housed in outdoor hutches in a continental winter. Further research on this concept would be beneficial, including in other regions or housing systems, or to evaluate outcomes relating to immune function or health using a larger sample size.

**Figure 1:**
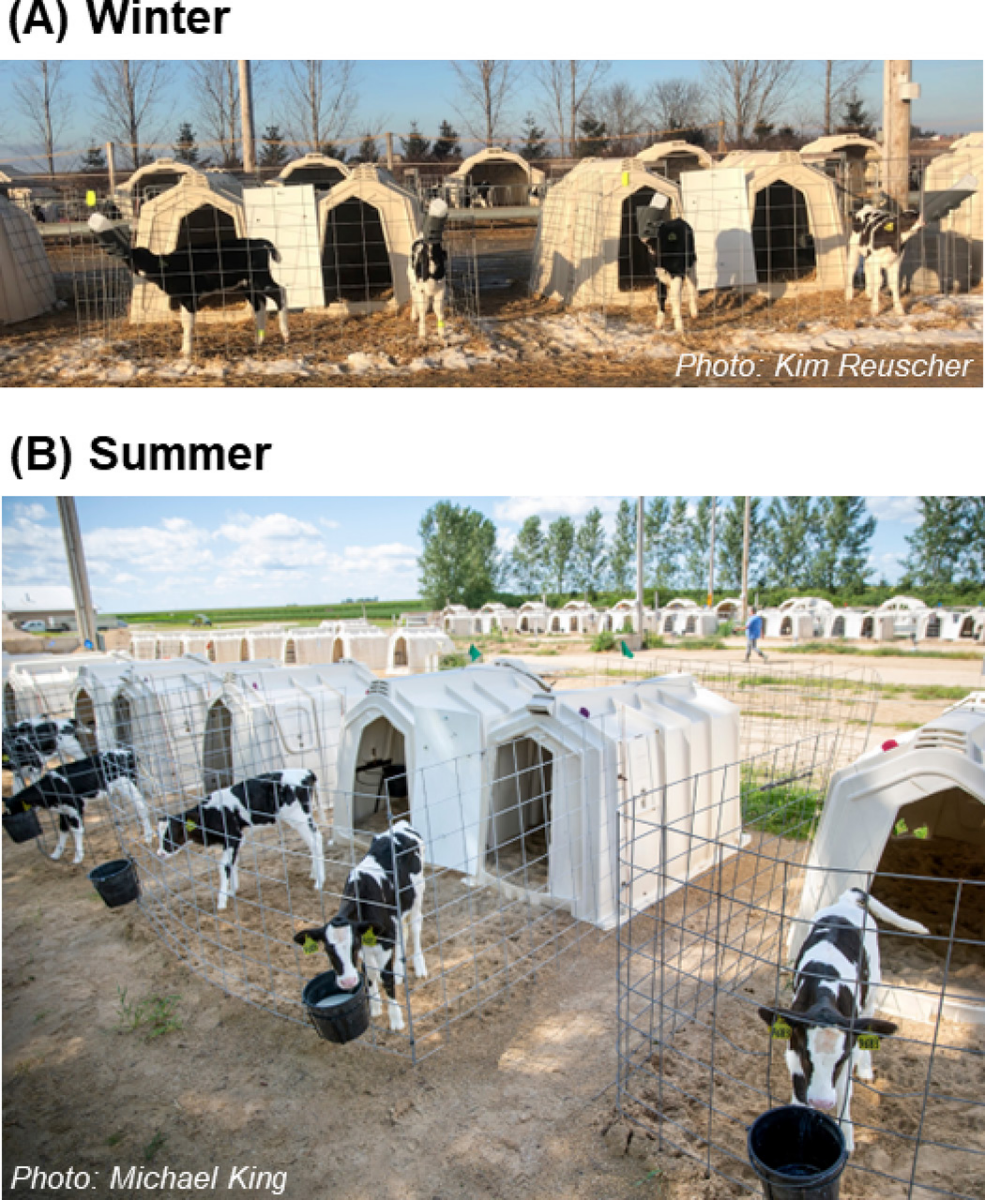
Calves were housed in outdoor hutches in (A) winter and (B) summer in a continental climate. Pair-housed calves (both seasons) were provided with 2 polyethylene hutches (each 1.1 × 2.1 m inside) connected with wire fencing (2.7 × 1.8 m area). For individually housed calves (winter only), the outdoor area was 1.2 × 1.8 m.

**Figure 2:**
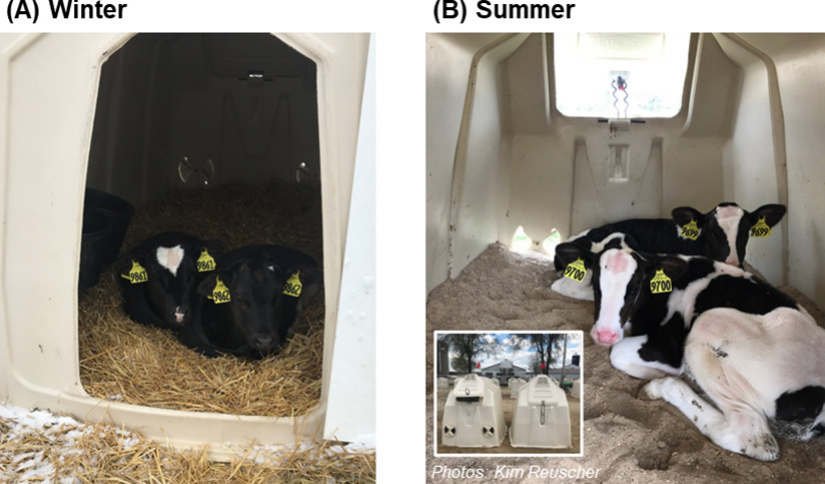
Pair-housed calves had 2 hutches and preferred to spend time together in the same hutch in both (A) winter and (B) summer. The heat load inside the hutch was greater with 2 calves versus 1 calf inside. In (B) summer, additional passive ventilation from rear openings (inset, panel B), which could be closed in winter to prevent drafts, mitigated this effect; calves preferred to spend time in the ventilated hutches.

For the same reasons calves are more susceptible to cold stress compared with older cattle, they have been traditionally thought to be less vulnerable to heat stress. This perception is, in part, because lactating cows produce considerable metabolic heat (approximately twice as much as when nonlactating; Bianca, 1968). The effects of heat stress on lactating cows, including significant decreases in milk production, are well documented and receive greater attention in part due to the downstream economic consequences of these impacts (St-Pierre et al., 2003). However, regardless of the calves' current or future contributions to a farm's profit, their present welfare has inherent value. More research has begun to evaluate heat stress and strategies for its abatement in calves. This topic is timely and critical, with climate change models predicting an increase in global average temperatures and more frequent heat waves (IPCC, 2022). Recent studies by our groups have identified temperature-humidity index (**THI**; NRC, 1971) breakpoints of 65 and 69 for calves in US subtropical versus continental climate zones, respectively (Dado-Senn et al., 2020a, 2023). These breakpoints were based on abrupt changes in respiration rate (**RR**), an early thermoregulatory coping response and common indicator of heat stress in cattle. Notably, those breakpoints for calves are similar to those for adult cows (THI = 65 or 70, depending on posture, in a continental climate; Pinto et al., 2020); this demonstrates that the assumption that calves are less susceptible to heat-related discomfort is a faulty one.

Studies have documented negative impacts of heat stress on biological functioning, including reduced starter grain intake (López et al., 2018), impaired ADG (Hill et al., 2011), reduced serum IgG (Marrero et al., 2021), and greater rates of morbidity (Louie et al., 2018) and mortality (Stull et al., 2008). Heat stress mitigation strategies depend on whether calves are housed in outdoor hutches or a barn. While barn roofs provide shade, outdoor hutches, depending on their material, can create a greenhouse effect, with internal temperatures greater than ambient (Lammers et al., 1996). To reduce heat gain from solar radiation in outdoor hutches, strategies have included providing supplemental shade over the hutches (Kovács et al., 2018) or reflective covers (Carter et al., 2014). To promote heat dissipation, passive ventilation strategies include elevating the hutches (Moore et al., 2012) or adding openings to increase air exchange (Reuscher et al., 2019). In barns, mechanical ventilation such as fans provide forced air movement to assist calves with directly dissipating heat (Hill et al., 2011; Dado-Senn et al., 2020b, 2022). These heat abatement strategies have been shown to improve biological functioning, as measured by thermoregulatory coping responses (e.g., RR, rectal temperature) as well as milk and starter intake and health indicators (Dado-Senn et al., 2020b).

In terms of behavioral indicators of thermal preference or attempts to alleviate thermal discomfort, group-housed calves in barns with overhead fans spent more time lying in the center of the pen, where air speed was the greatest (subtropical climate; Dado-Senn et al., 2022). Another recent study by our group was the first to evaluate calves' preferences for heat abatement (continental climate; Reuscher et al., 2024b). Using outdoor paired hutches (within each pair of hutches, one had additional passive ventilation; Figure 1B), we evaluated how social proximity and passive hutch ventilation interacted to affect calf heat stress responses. With additional openings on the back of the hutch (which could be closed in winter to reduce drafts; Figure 3), the greater air exchange reduced the internal hutch microclimate THI and calves' RR after 1 h inside, compared with when the rear openings were closed. Calves contributed greater heat load to the internal hutch microclimate as they aged, but passive ventilation mitigated this effect. Calves preferred to spend 80% of their time together, including inside the nonventilated hutch, which suggested their desire for social proximity outweighed the negative thermal effects of sharing a nonventilated hutch. Nonetheless, calves preferred spending time in the ventilated hutch (in wk 6 and 9 of life, but not wk 4, when they used both hutch types equally; Reuscher et al., 2024b), demonstrating they sought out a more thermally comfortable environment.

Passively ventilating outdoor hutches is a relatively simple and low-cost strategy to increase air exchange and reduce heat stress in calves, including those housed in pairs. However, this strategy depends on outside air movement and does little to increase air speeds inside the hutch, limiting direct convective cooling of the calves. Recently, we developed a novel method for mechanically ventilating outdoor hutches, using solar-powered fans to blow air into the rear of the hutch. These fans showed promise for reducing internal hutch temperature and calves' RR and rectal surface temperatures compared with nonventilated or passively ventilated hutches (Dado-Senn et al., 2024), but this method is experimental and not yet economically viable for commercial-scale use. Furthermore, the fans have been tested only with individually housed calves; future research could examine the preferences of pair-housed calves for using mechanically ventilated hutches.

Together, our continental summer and winter studies of calves housed in outdoor hutches showed that pair housing has some potential for protecting against cold stress, and that simple, passive hutch ventilation can mitigate the negative effects of heat stress when calves spend time together inside a hutch. Our findings also support previous evidence that calves prefer and are motivated for social proximity. Additional unexpected results for ocular temperature (**OT**) from these studies seemed to underscore the importance of social contact for calf welfare. Ocular temperature has been used as an indicator of stress in cattle, including thermal stress (Scoley et al., 2019). We had predicted OT would be greater when pair-housed calves were restricted inside a hutch together (due to sharing heat load), compared with when they were in separate hutches or when compared with individually housed calves, but we found the reverse patterns (Reuscher et al., 2024a,b). We hypothesize the visual isolation from conspecifics may have increased OT as a stress response, and this variable may have unintentionally served as a physiological measure of the calves' affective states, rather than simply an indicator of thermoregulation.

To date, no studies have directly evaluated the affective states of dairy cattle, let alone calves, in the context of thermal stress. An increasingly used technique across species is the judgment bias test, in which more optimistic versus pessimistic responses to ambiguous stimuli are interpreted to represent more positive versus negative affective states, respectively (Bučková et al., 2019). However, for preweaning calves, milk or milk replacer is typically used as the rewarding stimulus; because appetite decreases or increases as an adaptive response to heat or cold stress, respectively, this presents a methodological challenge and potential confound when evaluating affective states (or cognitive performance; Dado-Senn et al., 2020c) in the context of thermal stress. Nonetheless, we encourage future research to address the major knowledge gap on how thermal stress and mitigation strategies impact calves' affective states.

Indirect inferences about affective states can be made by evaluating preferences and motivations (Kirkden and Pajor, 2006). A few studies have documented calves' seeking of heat in winter (Borderas et al., 2009) and high-speed air from fans in summer (Dado-Senn et al., 2022), and their preferences for outdoor hutches with greater passive air exchange (Reuscher et al., 2024b). However, knowledge gaps remain regarding calves' preferences for heat abatement resources such as shade or water spray, and no studies have evaluated their motivation for accessing either heat- or cold-stress abatement resources. For example, lactating cows are motivated to access shade (Schütz et al., 2008), and they prefer this resource compared with direct sun exposure, including unshaded sprinklers, despite the latter's effectiveness for reducing thermoregulatory indicators of heat stress (Schütz et al., 2011). Although studies have demonstrated biological functioning benefits of providing supplemental shade over outdoor hutches (Kovács et al., 2018), future research evaluating calves' preferences and motivations for shade could potentially bolster the importance of this resource from an animal welfare perspective.

In addition, studies on older cattle have demonstrated that direct soaking with low-pressure water spray provides effective cooling across climate regions (Van Os, 2019). Soakers not only improve biological functioning, as measured by both thermoregulatory and production responses, but also are preferred by lactating cows (Chen et al., 2013) and 10-mo-old beef steers (Parola et al., 2012) in combination with shade, as compared with shade alone. An alternative is the use of high-pressure misters or foggers. Although this method cools the microclimate only in lower humidity climates, it has the upside of not wetting the bedding or feed, as the fine droplets evaporate before landing. Although water lines for soaker or mister systems are likely impractical for outdoor hutches, these methods could potentially be used in some calf barns. Future research could evaluate water spray systems for cooling calves, including physiological, health, production, and behavioral outcomes.

In conclusion, cold and heat stress present welfare challenges for dairy calves, particularly in the context of global climate change. Many studies have demonstrated the consequences of thermal stress on biological functioning, and several strategies have been evaluated to mitigate those detrimental effects. Nonetheless, knowledge gaps remain regarding impacts on calves' affective states and their preferences and motivations for specific thermal stress mitigation resources. Furthermore, additional research is needed to evaluate the intersection of thermal stress and other factors important to calf welfare, such as social contact. Research to address these gaps could improve our understanding of calf welfare and inform best management practices.
